# The draft genome sequence of *Diaporthe vaccinii*, isolated from diseased cranberries

**DOI:** 10.1128/mra.00466-24

**Published:** 2025-04-02

**Authors:** Bhagya C. Thimmappa, Matt Sarrasin, B. Franz Lang, Gertraud Burger

**Affiliations:** 1 Department of Biochemistry, Robert-Cedergren Center for Bioinformatics and Genomics, Université de Montréal 5622, Montreal, Québec, Canada; University of Strathclyde, Glasgow, United Kingdom

**Keywords:** fruit-rot pathogen, nuclear genome, *Diaporthe vaccinii*, phytopathogen, *Vaccinium macrocarpon*

## Abstract

We report the assembly and annotation of the nuclear genome of *Diaporthe vaccinii*, a pathogenic fungus isolated from diseased cranberries in Quebec, Canada. The genome was sequenced with the Illumina paired-end sequencing technology, assembled into 67 Mbp across 588 contigs, with an N50 of 386 Kbp and 97.5% BUSCO completeness.

## ANNOUNCEMENT

The North American cranberry is economically important but susceptible to fungal pathogens, collectively called the cranberry fruit rot (CFR) complex, causing significant crop losses (1–3). One CFR genus is *Diaporthe*, with the species *Diaporthe vaccinii* (anamorph *Phomopsis vaccinii*) causing, for example, stem cankers and soft rot in blueberries (4) and cranberries (*Vaccinium* spp.) (5–8).

Our previous work showed that the *D. vaccinii* isolate IS7, isolated from diseased cranberries, infects cranberry plants and leads to their death (9). Here, we report a high-quality draft genome of *D. vaccinii* IS7 that will facilitate further investigation of its pathogenicity.

The sample was collected by Richard Bélanger (Université Laval, Québec, Canada) in 2013 from the flesh of diseased cranberries in Saint-Louis-de-Blandford (Quebec-Canada) using the hyphal tip isolation method and kindly provided to us. The isolate was grown on potato dextrose agar medium and maintained at 4°C with subculturing every 6 months. For genome sequencing, IS7 was cultured on yeast-glycerol medium (YGM; yeast extract 0.4%, glycerol 2%, and pH 7) at room temperature for 3 days and disrupted in a blender (Hamilton Beach, model-51109C, 225 W for 3 min). DNA was isolated using the Qiagen DNeasy PowerPlant Pro Kit (Qiagen-GmbH) according to the manufacturer’s protocol. Taxonomic identification was performed by PCR amplification of the Internal Transcribed Spacer (ITS) region (ITS1-5.8S-ITS2) using the fungus-specific primers BMB-CR forward 5ʹ-GTACACACCGCCCGTCG-3ʹ (binds small-subunit rRNA) and ITS4 reverse 5ʹ-TTCCWCCGCTTATTGATATGC-3ʹ (binds large-subunit rRNA) (10); the PCR product was purified using the QIAquick Gel Extraction Kit (Qiagen-GmbH) and Sanger sequenced by the Institut de Recherche en Immunologie et en Cancer (IRIC) Genomics Platform (Montreal-Canada) using the above primers. A blastn (v2.16.1) search (11) against the National Center for Biotechnology Information (NCBI) non-redundant database (update date: 30 December 2024) assigned IS7 to *D. vaccinii* (GenBank accession PP921214.1) with 100% identity and coverage.

For whole-genome sequencing, liquid YGM was inoculated with ~1 × 10^6^ Colony-Forming Units (CFUs) of an IS7 hyphal suspension. The culture was grown under shaking for 3 days at room temperature. Genomic DNA was isolated using the DNeasy Plant Mini Kit (Qiagen-GmbH) according to the manufacturer’s recommendation. Library preparation (KAPA-HyperPrep Kit, Roche-Switzerland) and Illumina MiSeq paired-end sequencing with a 300 bp read length were outsourced to the Genome Quebec Center in Montreal.

Reads were trimmed of adapters using Trimmomatic (v0.35) (12) and corrected with Rcorrector (v1.0.4) (13). Default parameters were used for all software unless specified. The genome was *de novo* assembled using the SPAdes assembler (v3.14.1) (14). Structural genome annotation was performed using an in-house pipeline, which masks repeats and searches protein sequence similarity in UniProtKB and RefSeq (downloaded November 2017) employing Spaln (v2.2.2) (15). The *ab initio* predictors used were Augustus (v3.3.2) (16), Snap (17), Genemark (v4.33) (18), and CodingQuarry (v2.0) (19). Functional assignments of protein-coding genes were performed by profile Hidden Markov Model (HMM) searches with HMMER3 (v3.4) (20) in UniProtKB/Swiss-Prot release 2022_02 and PFAM database (v37.1) (21). Genome assembly completeness was assessed with BUSCO (v5.1.0) (22) using the OrthoDB (v10) Sordariomycetes data set (23). Details of the assembly are compiled in Table 1.

**TABLE 1:** Nuclear genome statistics of *D. vaccinii* IS7

Genome feature	
Nr. of raw reads	16,891,320
Raw read length	300 bp
Estimated coverage	25×
Size of the assembly	67,097,566 bp
Nr. of contigs (>200 bp)	588
N50	386 Kbp
Size of the largest contig	1,775,149 bp
G + C content	46.4%
Genome completeness (BUSCO)	97.5%
Nr. of protein-coding genes	15,650
Mean gene length	2,099 bp
Mean exon length	736 bp
Annotation completeness (BUSCO)	99.8%

## Data Availability

Genome sequence reads (SRR28883308) and RNA-Seq data (SRR28883305, SRR28883306, and SRR28883307) were deposited in the Sequence Read Archive, and the Whole-Genome Shotgun project (JBAWTH000000000) at DDBJ/ENA/GenBank under BioProject PRJNA1084027. The version described in this paper is JBAWTH010000000.
